# Presentation, Management, and Outcomes Across the Rural-Urban Continuum for Hepatocellular Carcinoma

**DOI:** 10.1093/jncics/pkaa100

**Published:** 2020-11-02

**Authors:** Kali Zhou, Trevor A Pickering, Christina S Gainey, Myles Cockburn, Mariana C Stern, Lihua Liu, Jennifer B Unger, Anthony B El-Khoueiry, Norah A Terrault

**Affiliations:** 1 Department of Medicine, University of Southern California, Los Angeles, CA, USA; 2 Department of Preventive Medicine, University of Southern California, Los Angeles, CA, USA; 3 Norris Comprehensive Cancer Center, University of Southern California, Los Angeles, CA, USA

## Abstract

**Background:**

Hepatocellular carcinoma is 1 of few cancers with rising incidence and mortality in the United States. Little is known about disease presentation and outcomes across the rural-urban continuum.

**Methods:**

Using the population-based Surveillance, Epidemiology, and End Results registry, we identified adults with incident hepatocellular carcinoma between 2000 and 2016. Urban, suburban, and rural residence at time of cancer diagnosis were categorized by the Census Bureau’s percent of the population living in nonurban areas. We examined association between place of residence and overall survival. Secondary outcomes were late tumor stage and receipt of therapy.

**Results:**

Of 83 368 incident cases of hepatocellular carcinoma, 75.8%, 20.4%, and 3.8% lived in urban, suburban, and rural communities, respectively. Median survival was 7 months (interquartile range = 2-24). All stage and stage-specific survival differed by place of residence, except for distant stage. In adjusted models, rural and suburban residents had a respective 1.09-fold (95% confidence interval [CI] = 1.04 to 1.14; *P* < .001) and 1.08-fold (95% CI = 1.05 to 1.10; *P* < .001) increased hazard of overall mortality as compared with urban residents. Furthermore, rural and suburban residents had 18% (odds ratio [OR] = 1.18, 95% CI = 1.10 to 1.27; *P* < .001) and 5% (OR = 1.05, 95% CI = 1.02 to 1.09; *P* = .003) higher odds of diagnosis at late stage and were 12% (OR = 0.88, 95% CI = 0.80 to 0.94; *P* < .001) and 8% (OR = 0.92, 95% CI = 0.88 to 0.95; *P* < .001) less likely to receive treatment, respectively, compared with urban residents.

**Conclusions:**

Residence in a suburban and rural community at time of diagnosis was independently associated with worse indicators across the cancer continuum for liver cancer. Further research is needed to elucidate the primary drivers of these rural-urban disparities.

Hepatocellular carcinoma (HCC) incidence in the United States increased by 32%, and deaths increased by 25% over the last 10 years (1). In 2010, 19% of Americans, numbering nearly 60 million, lived in rural areas (2). Similar to other cancers, incidence of HCC is slightly lower in rural compared with urban communities (6.3 vs 7.9 per 100 000 persons) (3); however, rural-urban differences in HCC stage at presentation, treatment patterns, and mortality are not well-characterized, yet they may have implications for healthcare policy and resource allocation (4).

Many studies have reported lower cancer survival in rural vs urban settings (5,6), a gap that may be widening (7). Rural Americans face unique challenges with respect to healthcare access and utilization, including higher rates of uninsured and underinsured, more poverty, reduced access to high-quality care, and greater distance to specialty services (8). Among patients with HCC eligible for transplant, distance to a liver transplant center has been negatively associated with being wait-listed for transplant, receiving transplant, and, ultimately, survival (9). An assessment of HCC presentation and outcomes across the rural-urban continuum is critical to quantifying remaining gaps in care, informing interventions at the community level, and providing a baseline for future comparison. HCC also differs from other cancers in its vulnerable at-risk population, distinct surveillance guidelines, and complex and multidisciplinary treatment decision making (10), which may exacerbate the divide in rural-urban outcomes. Racial and ethnic disparities are also commonly reported in HCC outcomes (11,12) but have not been evaluated across the rural-urban continuum despite differences in racial and ethnic composition.

We addressed these knowledge gaps with a comprehensive examination of the relationship between place of residence at diagnosis (urban, suburban, and rural) and HCC stage, receipt of therapy, and overall survival over the last 2 decades (2000-2016) using the population-based Surveillance, Epidemiology, and End Results (SEER) database. We additionally characterized temporal trends in stage and treatment by place of residence.

## Methods

### Case Selection

Incident HCC cases in SEER-18 registries, which cover up to 35% of the US population, diagnosed between January 1, 2000, and December 31, 2016, were included. HCC was defined using International Classification of Diseases for Oncology, 3rd edition topography code C22.0 and restricted to histology codes 8170-8175. All cases older than 18 years of age were included. Those with missing or insufficient residential data for rural-urban classification (overall missing = 5.6%) were excluded.

### Rural-Urban Classification

We classified rural-urban residence using the Census Bureau’s percent of the population living in nonurban areas, which consists of 4 categories on a continuum: 100% urban, 50% or more but less than 100% urban, more than 0% but less than 50% urban, and 100% rural tracts. The Census Bureau employs a uniform set of rules based on residential density, land use, distance, and population threshold to determine urban tracts; all other areas are deemed rural (2). Cancers diagnosed from 2000 to 2005 were linked to rurality variables estimated at year 2000 and cancers diagnosed in 2006-2016 linked with rurality variables estimated at year 2010 in the SEER database. We collapsed the 4 categories into 3 groups for our primary predictor of interest: “urban” as 100% urban, suburban as either 50% or more but less than 100% urban or more than 0% but less than 50% urban, and rural as 100% rural tracts.

### Study Covariates and Outcomes

Race and ethnicity were categorized as Hispanic (all races), non-Hispanic White, non-Hispanic Black, non-Hispanic Asian or Pacific Islander, non-Hispanic American Indian or Alaska Native, and unknown. Hispanic ethnicity was coded using a standardized identification algorithm (13). Insurance status was grouped as insured (including private and Medicare), Medicaid, uninsured, and unknown. We used the census-tract level Yost socioeconomic (SES) index, categorized into tertiles (low, middle, high) (14,15). Indices were linked to cancer cases by census tract and year of cancer diagnosis (16). Tumor stage was categorized as localized (confined to liver), regional (either direct extension or lymph node involvement), distant (metastatic disease), and unstaged. First course of therapy within 6 months of diagnosis is routinely collected by SEER. HCC therapy was categorized as surgery only, radiation only, chemotherapy only, combination treatment (more than 1 type received), or no treatment or unknown. US region (West, Northeast, Midwest, and South) was defined by the Census Bureau (17).

Primary outcome was overall survival. Mortality data from the National Center for Health Statistics were updated to December 31, 2017. Secondary outcomes included late stage at diagnosis and receipt of therapy. Late stage was defined as regional, distant, and unstaged and receipt of therapy as any therapy.

### Statistical Analysis

Demographic and tumor characteristics were compared by place of residence using descriptive χ^2^ and Wilcoxon rank sum testing as appropriate. Trends in proportion of individuals with given HCC stage at diagnosis and each treatment type between 2000 and 2016 were stratified by place of residence using linear regression. A linear term for year was sufficient for all groups except receipt of chemotherapy, because the number of individuals who received chemotherapy increased sharply between 2005 and 2010. Therefore, for proportion of individuals receiving chemotherapy, year was modeled as a dichotomous variable to reflect proportion before and after the increase. An iterative approach was used to identify the best cut-point for year (2007), and the cut-point was retained to reflect the effect of time in this model.

Kaplan-Meier survival curves were presented stratified by place of residence. Cox regression was performed with inclusion of univariate variables with *P* less than  .10 in multivariate models to examine the association between rural-urban classification and primary outcome of survival. Multivariate logistic regression was used for secondary outcomes of late stage and receipt of therapy (as binary outcomes). Because insurance data was only available after 2007, secondary analyses for all outcomes were performed with exclusion of cases diagnosed prior to 2007 and inclusion of insurance variable in models. Analyses were performed in R version 3.6.1. (Vienna, Austria) and Stata version 14.2 (College Station, TX).

## Results

### Characteristics of Adults With HCC Across Rural-Urban Continuum

There were 83 368 incident cases of HCC diagnosed in adults between the years 2000 and 2016. The majority (75.8%) were diagnosed in urban communities, followed by suburban (20.4%) and rural (3.8%) communities. Sociodemographic and tumor characteristics stratified by rural-urban classification are presented in Table 1. Median age at diagnosis was 63 years (interquartile range [IQR] = 56-72 years) for all 3 groups (*P* = .57). Sex, race and ethnicity, marital status, insurance, and SES differed statistically significantly across place of residence (all *P* < .001). Rural HCC cases were least likely to be in the highest SES tertile (14.7% of rural vs 25.2% of urban and 33.2% of suburban patients; *P* < .001). Of the HCC cases, 48.0%, 47.2%, and 43.9% were diagnosed at localized stage in urban, suburban, and rural communities, respectively (*P* < .001). A respective 45.4%, 45.4%, and 48.5% received no treatment within 6 months of diagnosis (*P* < .001). Among treated cases, chemotherapy was the most common treatment for all groups, followed by surgery, combination therapies, and radiation.

**Table 1.** Characteristics of HCC cases across rural-urban continuum

Characteristic	Overall (n = 83 368)	Urban (n = 63 204)	Suburban (n = 16 971)	Rural (n = 3193)	*P*
Median age, y (IQR)	63 (56-72)	63 (56-72)	63 (56-72)	63 (56-72)	.57
Age group, y, %					.001
<40	1.5	1.5	1.3	1.0	
40-49	6.8	7.0	6.6	5.8	
50-59	20.1	29.0	29.3	29.4	
60-69	32.3	31.0	32.1	32.9	
≥70	31.3	31.5	30.6	30.9	
Sex, %					<.001
Male	76.2	75.6	77.7	79.0	
Female	23.9	24.4	22.3	21.0	
Race/ethnicity, %					<.001
NH White	49.9	43.0	69.6	84.1	
NH Black	13.3	14.9	8.8	6.6	
NH AI/AN	0.8	0.7	1.0	1.9	
NH Asian/API	17.0	20.2	8.0	1.5	
Hispanic	18.8	21.1	12.6	5.9	
Unknown	0.2	0.3	10.0	0.1	
Marital status, %					<.001
Married	51.2	50.1	54.8	53.9	
Not married	44.2	45.4	40.2	41.2	
Unknown	4.6	4.5	5.1	4.9	
Insurance, No.^a^	59 940	45 036	12 496	2408	<.001
Uninsured, %	3.9	3.7	4.2	5.7	
Medicaid, %	23.3	24.9	18.3	19.0	
Insured, %	67.7	66.5	71.8	68.7	
Unknown, %	5.1	4.9	5.7	6.6	
Census tract-level SES, %					<.001
Lowest tertile	37.9	40.3	29.1	38.3	
Middle tertile	34.2	32.9	37.0	45.7	
Highest tertile	26.4	25.2	33.2	14.7	
Unknown	1.4	1.6	0.7	1.4	
Tumor stage, %					<.001
Localized	47.7	48.0	47.2	43.9	
Regional	26.6	26.6	26.2	27.2	
Distant	14.9	14.7	15.2	16.1	
Unknown	10.9	10.7	11.3	12.8	
Receipt of treatment, %					<.001
None/unknown	45.5	45.4	45.4	48.5	
Surgery only	15.8	16.1	14.9	14.4	
Radiation only	3.6	3.5	4.0	4.2	
Chemotherapy only	25.2	25.3	25.0	23.6	
Combination	9.9	9.7	10.7	9.4	
US region, %					<.001
West	61.2	66.8	46.4	29.6	
Midwest	7.8	7.6	6.1	14.5	
South	18.0	11.8	34.5	53.6	
Northeast	13.3	13.9	13.1	2.3	
Alive, %	22.2	22.6	21.4	19.9	<.001
Cause of death, No.^b^	66 963	50 534	13 809	2620	<.001
HCC, %	71.3	71.2	71.2	73.7	
Other cause, %	11.9	12.1	11.1	10.5	
Missing/unknown, %	16.9	16.7	17.7	15.7	

a Insurance data available after 2007 in SEER. AI/AN = American Indian/Alaska Native; API = Asian Pacific Islander; HCC = Hepatocellular carcinoma; IQR = interquartile range; NH = non-Hispanic; SEER = Surveillance, Epidemiology, and End Results; SES = socioeconomic status

b HCC cases in which HCC was not the first reported tumor in SEER were excluded (n = 10 251).

### Intersection of Race and Ethnicity, Stage and Treatment, and Rural-Urban Continuum

**Figure 1.**
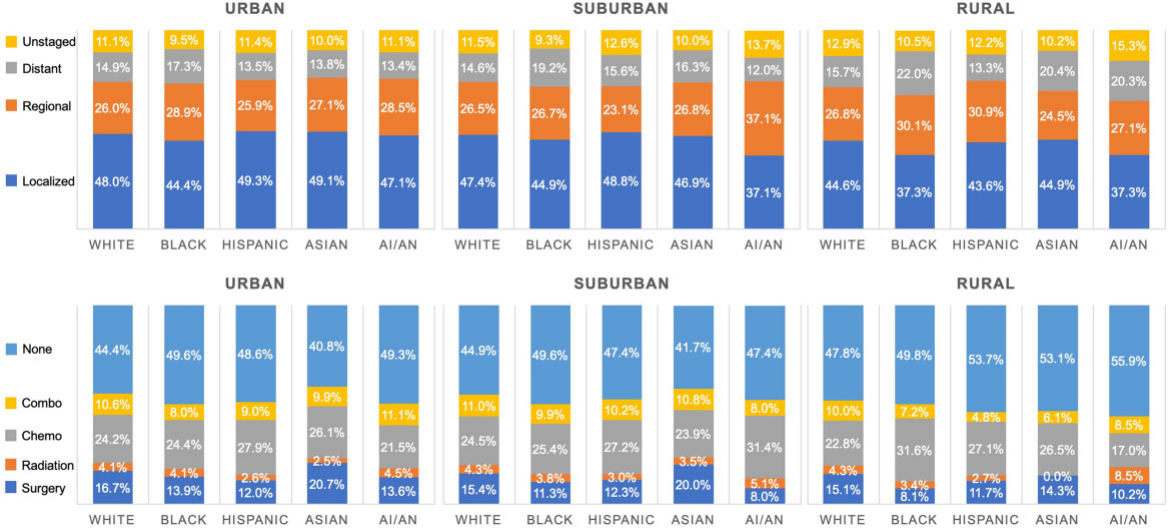
Race and ethnicity, stage, and receipt of therapy stratified by place of residence. AI/AN = American Indian/Alaskan Native

Differences in stage and treatment were seen by race and ethnicity across rural-urban continuum (Figure 1). Of HCC patients in rural communities, 84.1% were White, compared with 69.6% in suburban and only 43.0% in urban communities. Rural communities across racial and ethnic groups had lowest frequency of localized HCC: lowest for rural Blacks and non-Hispanic American Indian or Alaska Native at 37.3%, compared with 49.3% for urban Hispanics and Asians. Blacks had the lowest frequency of localized disease and highest of distant disease for all groups. No treatment was highest in rural communities across racial and ethnic groups, and surgical treatment was disproportionately higher in both rural and urban Asians and Whites. Lowest frequency of surgical treatment was seen in rural Blacks (8.1%), who also had the highest frequency of chemotherapy utilization (31.6%).

### Temporal Trends in HCC Stage and Treatment Rates

**Figure 2.**
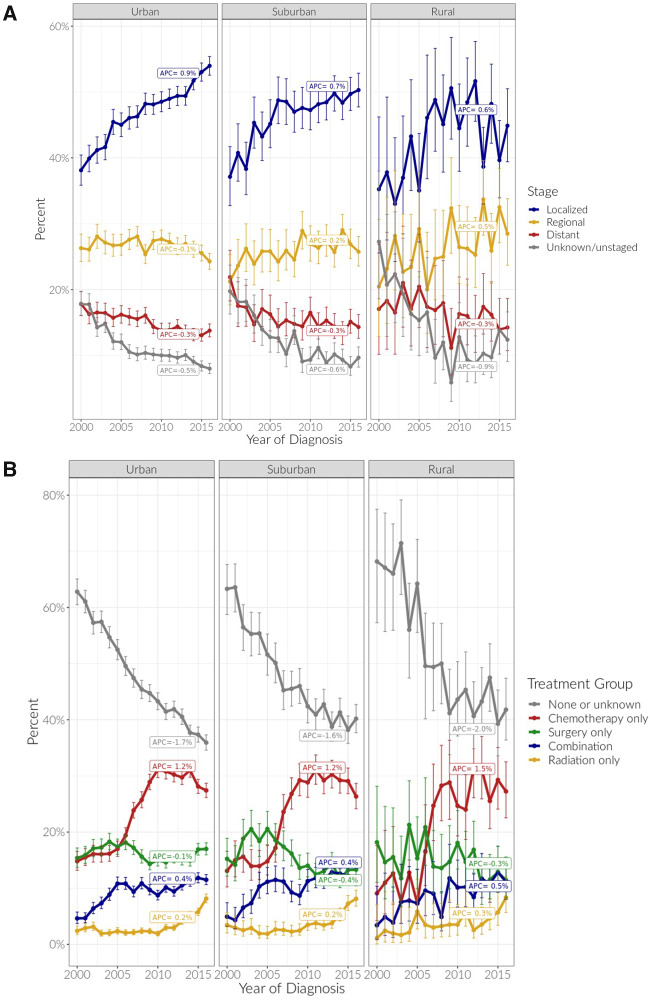
Trends in **(A)** stage at diagnosis and **(B)** treatment category over 2000-2016 time period by place of residence. The annual percent change (APC) is defined as the average annual percent change between 2000 and 2016.

We examined trends in stage at diagnosis and treatment over a 17-year time period (Figure 2;  Supplementary Tables 1 and 2, available online). Across the continuum, the proportion of individuals diagnosed with distant ($\beta_{\mathrm{year}}=$-0.26; *P* < .001) and unstaged disease ($\beta_{\mathrm{year}}=$0.53; *P* < .001) decreased, whereas localized stage increased over time ($\beta_{\mathrm{year}}=$+0.87; *P* < .001). For rural and suburban residents, proportion diagnosed with regional stage was initially lower than urban. However, whereas urban communities maintained a steady proportion diagnosed at regional stage, there was an increasing trend for rural and suburban communities (*P*_interaction_ = .05). Receiving no treatment decreased over time for all groups ($\beta_{\mathrm{year}}$ = -1.67; *P* < .001). Proportion receiving surgery only remained stable ($\beta_{\mathrm{year}}$ = -0.08; *P* = .48). Receipt of chemotherapy increased sharply around 2007 for all groups but less so for suburban vs urban communities (*P*_interaction_ = .01).

### Impact of Rural-Urban Continuum on Survival

**Figure 3.**
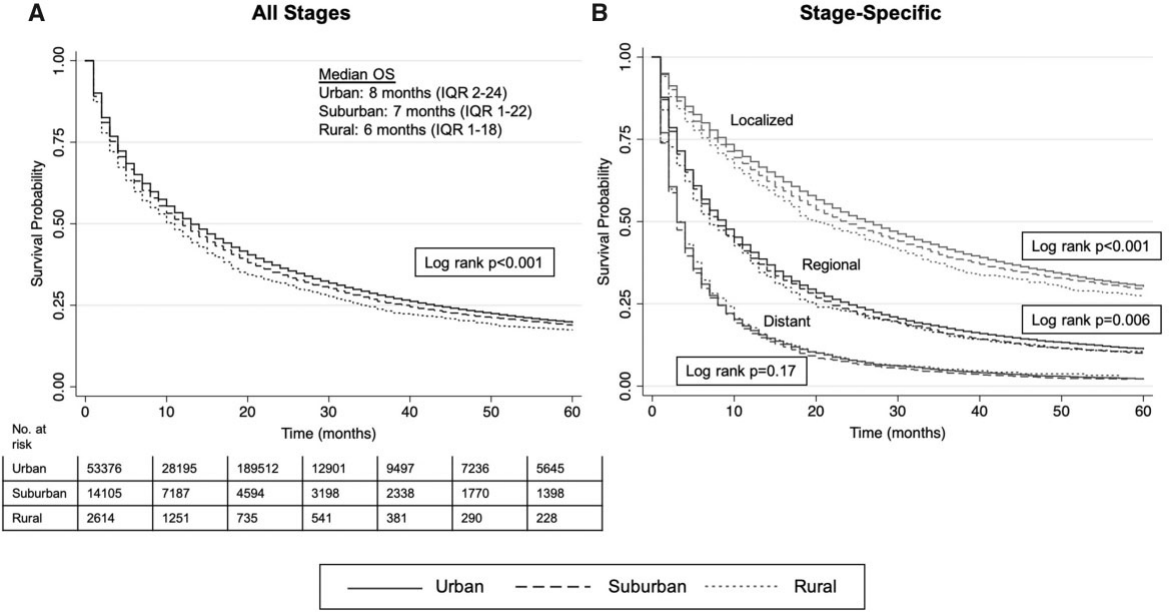
Kaplan-Meier curves for **(A)** overall and **(B)** stage-specific survival by place of residence. A 2-sided log-rank test was performed for equality of survivor functions. IQR = interquartile range; OS = overall survival.

Of incident cases, 1325 (1.6%) were diagnosed on death certificate only. For all others, median overall survival was 7 months (IQR = 2-24): 8 months (IQR = 2-24), 7 months (IQR = 1-22), and 6 months (IQR = 1-18) in urban, suburban, and rural communities, respectively (*P* < .01). HCC accounted for 71.3% of deaths, whereas death was attributed to other causes in 11.9% or missing or unknown in 16.9%. A difference in all stage and stage-specific overall survival was seen by place of residence, except for distant stage (Figure 3). Five-year survival rates by rural-urban classification ranged between 27.3% and 30.3% for localized, 9.8% and 11.3% for regional, 2.0% and 2.8% for distant, and 7.1% and 9.0% for unknown stage (Supplementary Figure 1, available online). In multivariable Cox regression with adjustment for age, sex, race and ethnicity, marital status, census tract (CT)-level SES, stage, treatment status, and year of diagnosis, place of residence was associated with overall survival (see Table 2; full results in Supplementary Table 3, available online). Suburban HCC patients had a 1.08-fold (95% confidence interval [CI] = 1.05 to 1.10) and rural patients a 1.09-fold (95% CI = 1.04 to 1.14) increased hazard of death as compared with urban patients. These results remained similar in our secondary models with adjustment for insurance status.

**Table 2.** Association between place of residence and primary and secondary outcomes

Model	Late stage at diagnosis^a^		Receipt of any therapy^b^		Overall survival^c^	
	OR (95% CI)	*P*	OR (95% CI)	*P*	HR (95% CI)	*P*
Full cohort, No.	83 368		83 368		70 095	
Urban	Referent		Referent		Referent	
Suburban	1.05 (1.02 to 1.09)	.003	0.92 (0.88 to 0.95)	<.001	1.08 (1.05 to 1.10)	<.001
Rural	1.18 (1.10 to 1.27)	<.001	0.88 (0.80 to 0.94)	<.001	1.09 (1.04 to 1.14)	<.001
Cohort with insurance data, No.	59 940		59 940		51 155	
Urban	Referent		Referent		Referent	
Suburban	1.06 (1.02 to 1.10)	.008	0.92 (0.88 to 0.96)	<.001	1.10 (1.07 to 1.13)	<.001
Rural	1.15 (1.06 to 1.25)	.001	0.92 (0.84 to 1.01)	.08	1.11 (1.05 to 1.17)	<.001

a Model adjusted for age, sex, race and ethnicity, marital status, socioeconomic status, and year of diagnosis (continuous). CI = confidence interval; HR = hazard ratio; OR = odds ratio.

b Model adjusted for above and tumor stage at diagnosis.

c Model adjusted for above, tumor stage at diagnosis, and receipt of therapy.

### Association Between Place of Residence, Late Stage HCC, and Receipt of Therapy

Place of residence was associated with late-stage HCC and likelihood of receiving therapy in multivariable analyses (Table 2; full results in Supplementary Table 4, available online). HCC patients living in rural and suburban communities had 18% and 5% higher odds of being diagnosed at late stage (OR = 1.18, 95% CI = 1.10 to 1.27 for rural and OR = 1.05, 95% CI = 1.02 to 1.09 for suburban) compared with urban communities after adjusting for age, sex, race and ethnicity, marital status, CT-level SES, and year of diagnosis. HCC patients in rural and suburban communities were 12% (OR = 0.88, 95% CI = 0.80 to 0.94) and 8% (OR = 0.92, 95% CI = 0.88 to 0.95) less likely to receive any treatment, respectively, compared with urban patients, after adjusting for age, sex, race and ethnicity, marital status, CT-level SES, stage, and year of diagnosis. These relationships remained the same after adjustment for insurance status.

### Regional Differences in Survival Across Rural-Urban Continuum

The South had the highest proportion of cases (11.4%) from rural communities, compared with 7.3% in the Midwest, 1.9% in the West, and only 0.7% in the Northeast. For urban patients, residence in the Midwest (hazard ration [HR] = 1.12, 95% CI = 1.06 to 1.17) and the South (HR = 1.14, 95% CI = 1.10 to 1.19) were independently associated with increased hazard of death compared with the West (Supplementary Table 5, available online). Suburban (HR = 1.14, 95% CI = 1.08 to 1.21) and rural (HR = 1.26, 95% CI = 1.11 to 1.42) residents in the South also had worse survival compared with those in the West.

## Discussion

In this nationwide study of adults with incident HCC between 2000 and 2016, we demonstrated consistently worse indicators across the cancer continuum for HCC for nonurban communities. Patients living in rural and suburban communities at HCC diagnosis in the United States were more likely to be diagnosed at late stage and less likely to have received any type of treatment. All-cause mortality was higher among rural and suburban residents, despite accounting for more late-stage HCC, lower treatment uptake, and sociodemographic factors. Important next steps for investigation include the relative contribution of local cancer-related healthcare policies and awareness, access to specialists and treatments, and/or quality-of-care differences (18) to HCC-specific rural-urban disparities.

A complex interplay between residential location, race, and structural factors likely drives rural-urban healthcare disparities. One study on HCC found race and ethnicity to be a stronger predictor of survival than rural residency (19). Others highlight structural barriers to rural cancer care such as scarcity of services, insufficient public transportation, and retaining high-quality providers (8). Our study exemplifies this complexity: early stage diagnoses occurred least in rural Blacks (37.3%) yet most in urban Hispanics (49.3%), whereas nontreatment was highest in rural Hispanics (53.7%) and lowest in urban Asians (40.8%). However, the survival disparity by place of residence persisted when accounting for racial and ethnic composition. In a study that aggregated clinical trials of cancer therapies (20), no difference in survival by rural-urban residence was found, suggesting that standardizing treatment access and quality would improve outcomes in nonurban settings and simultaneously promote equity for disadvantaged racial and ethnic groups. Furthermore, a fragmented US healthcare system, rather than integrated or universal health care, may contribute to rural health disparities, as other countries that have adopted universal coverage have witnessed improvements in the rural-urban divide (21).

The largest gap in survival by place of residence occurred in those who presented with localized disease, as survival was uniformly poor at more advanced stages. Access to treatment may partially explain this gap. After adjustment for insurance, both rural and suburban communities were 8% less likely to receive treatment. Treatment of localized HCC is highly complex, with a wide range of available options among eligible (eg, surgical resection, targeted locoregional therapies, liver transplantation) with variable rates of cure. Even small differences in access to curative treatments may have an outsized impact on survival time (22); liver transplantation, in particular, is a scarce resource with the highest cure rates (approximately 80% at 5 years) yet largely performed in urban academic centers with distance a direct barrier to successful transplantation (9). Lack of detailed treatment history or characteristics of chronic liver disease in SEER limits our ability to draw conclusions on whether differences in treatment uptake by place of residence are driven by ineligibility for specific treatments, inadequate access, or both, and future studies linking registry patients to robust claims or treatment databases would provide valuable insight. Scarcity of specialty access has also been linked to higher mortality in rural Medicare beneficiaries (18) and may be particularly relevant for early stage HCC in which treatment decisions made by multidisciplinary teams and training centers translate into better survival (23,24). Enhanced strategies to increase access for rural and suburban patients to urban academic centers where multidisciplinary teams are available, such as multidisciplinary co-located clinics (25) or virtual case conferences and tumor boards (26), are needed.

Over the past 2 decades, more individuals were being diagnosed at localized stage across the rural-urban continuum, a promising trend. Most of the rise was accounted for by lower proportion of late and unstaged disease; however, suburban and rural communities had an increasing trend in regional stage at diagnosis, with relative stability in urban patients. We hypothesize that differences in HCC surveillance uptake may play a role in these trends. For example, Asians with hepatitis B are a targeted group for surveillance (10), and Asians are disproportionately represented in urban communities; higher likelihood of surveillance in this group may be one possible explanation. Increased uptake of treatment (any type) in the first 6 months was seen across the rural-urban continuum over time, primarily because of a sharp upsurge in systemic therapy uptake in 2007, corresponding to approval of sorafenib for treatment of advanced HCC (27,28). These trends are likely to continue with the introduction of immunotherapy and small molecule agents for advanced HCC in recent years (29,30). There is a vital need to better understand barriers to access across the rural-urban continuum to ensure all communities going forward benefit from advances in HCC therapeutics.

Regional variations in cancer survival are well described, including for HCC (31,32). The southern region of the United States is particularly vulnerable (33), as the region with highest rates of poverty, uninsured, and lowest density of physicians (34), and we demonstrate this to be the case across the rural-urban continuum for HCC survival. In fact, because the south is disproportionately rural, our findings are most consequential for this region. By state, the highest reported age-adjusted incidence of HCC is in Texas (35), related to a large Hispanic population with high prevalence of risk factors for liver disease. For this reason, research programs in Texas are underway to develop early detection as well as secondary and tertiary prevention strategies (36). Place of residence and associated geospatial characteristics should be incorporated into these programs to ensure developed strategies are efficacious and applied equitably to nonurban areas.

Limitations of this study include classification of rurality, defined here by census tract-level population density, which may not fully represent the “rural” population with respect to access to and utilization of health care. However, healthcare policies are typically created for and enacted along these administrative boundaries; thus, our definition serves a practical purpose. Prior comparative studies have found that different designations of rurality produce concordant findings (37). Generalizability to all rural communities across the United States is uncertain because of incomplete geographic coverage of SEER (38). We cannot evaluate the impact of discordance between place of residence and location of care, because rural residents, particularly those considering transplantation (9), may travel outside their immediate area to seek care. If occurring at high enough frequency, the true gap in rural-urban outcomes for HCC may be even larger. Secondly, although useful in providing a high-level overview, SEER lacks granularity with respect to stage, treatment, and underlying liver disease that is relevant to HCC. In particular, clinical staging of HCC incorporates severity of liver disease (Childs-Pugh score) and performance status, both important determinants of treatment eligibility. There are also potential residual confounders, related to the lack of clinical details, as well as the social and structural determinants of health that differ both between and within each place of residence. However, this is the first large population-based study to provide an evaluation of HCC care by rural-urban residence over the past 20 years, and we highlight some intriguing disparities that may pave the way for future research specific to individual geographic contexts.

In summary, the difference in the presentation, management, and survival of US adults with HCC across the rural-urban continuum is an underrecognized cancer disparity. Efforts to target early detection coupled with expansion of access to standardized treatment are important to improving HCC control in disadvantaged suburban and rural communities.

## Funding

This work was supported by a University of Southern California Research Center for Liver Diseases grant [5P30DK048522] to Dr Zhou.

## Notes

**Role of the funder:** The study sponsor had no role in the study design; in the collection, analysis, and interpretation of data; the writing of the manuscript; or the decision to submit the manuscript for publication.

**Disclosures:** Dr Zhou and Dr Terrault report institutional grant support from Gilead Sciences. The other authors reported no disclosures.

**Author contributions:** Kali Zhou is the guarantor of the article, and all authors approved the final version of the manuscript. The authors made the following contributions: Concept and design: Zhou, Terrault. Acquisition, analysis, or interpretation of data: Zhou, Pickering, Cockburn, Terrault. Statistical analysis: Zhou, Pickering. Drafting of the manuscript: Zhou. Critical revision of the manuscript for important intellectual content: All authors.

## Data Availability

The data underlying this article were accessed from SEER Census Tract-level SES and Rurality Database and is publicly available upon request: https://seer.cancer.gov/seertrack/data/request/. The derived data generated in this research will be shared on reasonable request to the corresponding author.

## Supplementary Material

pkaa100_Supplementary_Data
