# Supplementary material for: Presentation, Management, and Outcomes Across the Rural-Urban Continuum for Hepatocellular Carcinoma
Source: JNCI Cancer Spectr. 2020 Nov 2;5(1):pkaa100. doi: 10.1093/jncics/pkaa100 (PMC7791625; doi:10.1093/jncics/pkaa100)
Supplement: pkaa100_Supplementary_Data [file pkaa100_supplementary_data.pdf]

Supplementary Materials

Supplementary Table 1. Regression of year and place of residence on percent of individuals diagnosed in each stage

	Distant			Localized			Regional			Unknown/Unstaged		
**Predictors**	B	95%CI	p	B	95%CI	p	B	95%CI	p	B	95%CI	p
**Community Type (Ref=Urban)**												
**Rural**	-0.26	-0.41 – -0.12	0.001	0.87	0.56 – 1.19	<0.001	-0.08	-0.29 – 0.13	0.47	-0.53	-0.77 – -0.29	<0.001
**Suburban**	0.73	-1.18 – 2.63	0.46	0.89	-3.30 – 5.07	0.68	-3.53	-6.35 – -0.71	0.02	1.91	-1.31 – 5.13	0.25
**Year**	1.48	-0.43 – 3.38	0.14	-2.12	-6.31 – 2.07	0.33	-4.72	-7.54 – -1.90	0.002	5.36	2.14 – 8.58	0.002
**Year x Community Interaction**												
**Year x Rural**	-0.01	-0.22 – 0.19	0.89	-0.19	-0.64 – 0.25	0.40	0.33	0.03 – 0.63	0.04	-0.12	-0.46 – 0.22	0.50
**Year x Suburban**	0	-0.20 – 0.21	0.97	-0.23	-0.68 – 0.22	0.32	0.55	0.24 – 0.85	0.001	-0.32	-0.66 – 0.02	0.07

Supplementary Table 2. Regression of year and place of residence on percent of individuals diagnosed in each treatment type

	Chemotherapy Only			Combination			None or Unknown			Radiation Only			Surgery Only		
**Predictors**	B	95%CI	p	B	95%CI	p	B	95%CI	p	B	95%CI	p	B	95%CI	p
**Community Type (Ref=Urban)**															
**Rural**	-1.64	-4.12 – 0.85	0.20	0.09	-1.88 – 2.07	0.93	-0.6	-5.11 – 3.89	0.79	0.2	-1.44 – 1.88	0.79	1.81	-1.06 – 4.67	0.22
**Suburban**	-5.45	-7.93 – -2.97	<0.001	-1.6	-3.61 – 0.35	0.11	6.55	2.05 – 11.05	0.01	0.2	-1.47 – 1.85	0.82	0.58	-2.28 – 3.45	0.69
**Year**	12.26	9.98 – 14.55	<0.001	0.36	0.21 – 0.51	<0.001	-1.7	-2.01 – -1.33	<0.001	0.2	0.11 – 0.36	0.001	-0.1	-0.29 – 0.14	0.48
**Year x Community Interaction**															
**Year x Rural**	1.31	-1.93 – 4.55	0.43	0.08	-0.13 – 0.29	0.45	0.12	-0.36 – 0.60	0.63	0	-0.16 – 0.19	0.86	-0.3	-0.61 – 0.01	0.06
**Year x Suburban**	4.28	1.05 – 7.52	0.01	0.12	-0.09 – 0.33	0.27	-0.3	-0.76 – 0.20	0.26	0	-0.14 – 0.21	0.71	-0.2	-0.53 – 0.08	0.16

*For Chemotherapy Only, year is modeled as dichotomous ≥ 2007.

Supplementary Table 3. Full results of multivariable model for overall mortality

	Overall mortality N=70095		
	**OR**	**95% CI**	**p-value**
**Place of residence**			
Urban	ref		
Suburban	1.08	1.05-1.10	<0.001
Rural	1.09	1.04-1.14	<0.001
**Age group**			
<40	ref		
40-49	1.2	1.11-1.31	<0.001
50-59	1.31	1.21-1.42	<0.001
60-69	1.37	1.27-1.49	<0.001
>=70	1.73	1.60-1.87	<0.001
**Sex**			
Male	ref		
Female	0.89	0.87-0.91	<0.001
**Race/ethnicity**			
NH White	ref		
NH Black	1.06	1.03-1.09	<0.001
Hispanic	0.93	0.91-0.96	<0.001
NH Asian/API	0.83	0.81-0.86	<0.001
NH AI/AN	0.93	0.84-1.02	0.14
Unknown	0.46	0.36-0.59	<0.001
**Marital status**			
Not married	ref		
Married	0.91	0.89-0.93	<0.001
Unknown	0.82	0.79-0.86	0.33
**CT-level SES**			
Lowest tertile	ref		
Middle tertile	0.96	0.94-0.98	<0.001
Highest tertile	0.9	0.88-0.92	<0.001
Unknown	0.98	0.90-1.05	0.54
**Stage of disease**			

Localized	ref		
Regional	1.75	1.72-1.79	<0.001
Distant	2.93	2.85-3.01	<0.001
Unstaged	1.37	1.33-1.42	<0.001
**Treatment**			
None	ref		
Surgery only	0.27	0.26-0.28	<0.001
Radiation only	0.61	0.59-0.64	<0.001
Chemo only	0.58	0.56-0.59	<0.001
Multiple	0.32	0.31-0.33	<0.001

Supplementary Table 4. Full results of multivariable models for late stage at diagnosis and receipt of therapy

	Late stage at diagnosis N=83368			Receipt of therapy N=83368		
	**OR**	**95% CI**	**p-value**	**OR**	**95% CI**	**p-value**
**Place of residence**						
Urban	ref			ref		
Suburban	1.05	1.02-1.09	0.003	0.92	0.88-0.95	<0.001
Rural	1.18	1.10-1.27	<0.001	0.88	0.80-0.94	<0.001
**Age group**						
<40	ref			ref		
40-49	0.72	0.64-0.82	<0.001	0.59	0.51-0.68	<0.001
50-59	0.69	0.61-0.78	<0.001	0.59	0.52-0.67	<0.001
60-69	0.68	0.60-0.76	<0.001	0.59	0.52-0.67	<0.001
>=70	0.77	0.68-0.86	<0.001	0.34	0.30-0.89	<0.001
**Sex**						
Male	ref			ref		
Female	0.75	0.72-0.77	<0.001	1.11	1.07-1.15	<0.001
**Race/ethnicity**						
NH White	ref			ref		
NH Black	1.13	1.09-1.19	<0.001	0.86	0.82-0.90	<0.001
Hispanic	0.96	0.92-0.99	0.04	0.83	0.80-0.87	<0.001
NH Asian/API	0.98	0.94-1.02	0.3	1.1	1.05-1.14	<0.001
NH AI/AN	1.18	1.01-1.38	0.03	0.85	0.72-1.00	0.06
Unknown	0.98	0.73-1.32	0.92	0.79	0.57-1.09	0.15
**Marital status**						
Not married	ref			ref		
Married	0.86	0.84-0.89	<0.001	1.67	1.62-1.72	<0.001
Unknown	1.03	0.96-1.10	0.41	0.96	0.90-1.04	0.33
**CT-level SES**						
Lowest tertile	ref			ref		
Middle tertile	0.96	0.92-0.99	0.007	1.21	1.17-1.25	<0.001
Highest tertile	0.91	0.87-0.94	<0.001	1.47	1.41-1.53	<0.001
Unknown	0.91	0.81-1.02	0.12	1.1	0.97-1.24	0.15
**Stage of disease**	--					

Localized	--			ref		
Regional	--			0.53	0.51-0.54	<0.001
Distant	--			0.33	0.31-0.34	<0.001
Unstaged	--			0.12	0.11-0.13	<0.001

Supplementary Table 5. Association between US region and survival by place of residence

	Urban (n=38654)*			Suburban (n=10512)†			Rural (n=1989)‡		
**US Region**	**HR**	**95% CI**	**p-value**	**HR**	**95% CI**	**p-value**	**HR**	**95% CI**	**p-value**
West	ref	--		ref	--		ref	--	
Midwest	1.12	1.06-1.17	<0.001	1.09	0.98-1.21	0.12	1.09	0.91-1.30	0.35
South	1.14	1.10-1.19	<0.001	1.14	1.08-1.21	<0.001	1.26	1.11-1.42	<0.001
Northeast	0.98	0.94-1.01	0.24	1.05	0.97-1.13	0.23	0.88	0.60-1.29	0.53

*Model adjusted for age, sex, race/ethnicity, marital status, socioeconomic status, insurance, stage, treatment, and year of diagnosis
†Model adjusted for age, sex, race/ethnicity, marital status, socioeconomic status, insurance, stage, treatment, and year of diagnosis
‡Model adjusted for age, sex, marital status, insurance, stage, treatment, and year of diagnosis (race/ethnicity and socioeconomic status not significant in univariate model)

Supplementary Figure 1. 5-year survival rates by stage and residence

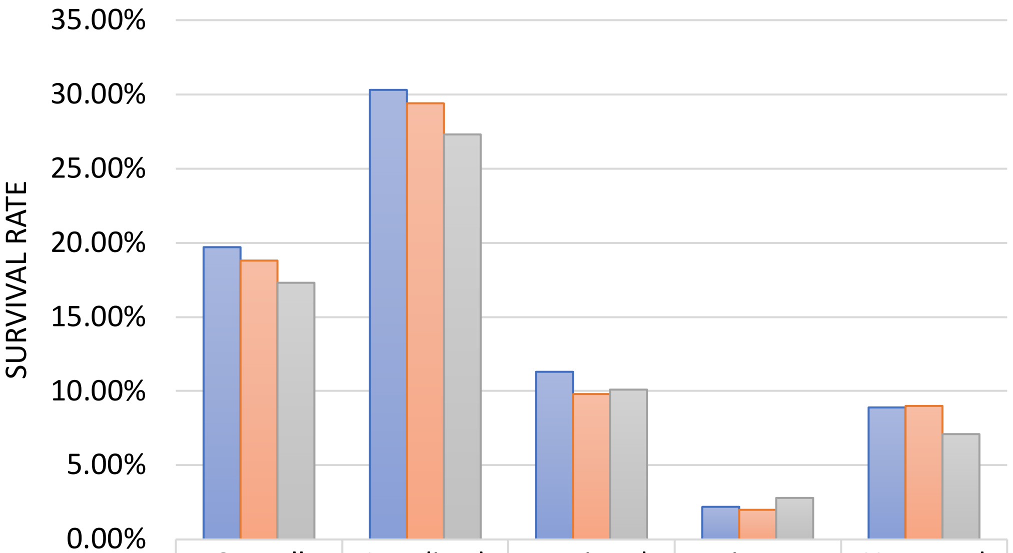

	Overall	Localized	Regional	Distant	Unstaged
Urban	19.70%	30.30%	11.30%	2.20%	8.90%
Suburban	18.80%	29.40%	9.80%	2.00%	9.00%
Rural	17.30%	27.30%	10.10%	2.80%	7.10%
